# The transcription factor Spores Absent A is a PKA dependent inducer of *Dictyostelium* sporulation

**DOI:** 10.1038/s41598-018-24915-w

**Published:** 2018-04-27

**Authors:** Yoko Yamada, Andrew Cassidy, Pauline Schaap

**Affiliations:** 1 School of Life Sciences, University of Dundee, Dundee, DD15EH Angus, UK; 2 Tayside Centre for Genomic Analysis, University of Dundee, Dundee, DD19SY Angus, UK

## Abstract

Sporulation in *Dictyostelium* fruiting bodies evolved from amoebozoan encystation with both being induced by cAMP acting on PKA, but with downstream components still being unknown. Using tagged mutagenesis to find missing pathway components, we identified a sporeless mutant defective in a nuclear protein, SpaA. Expression of prespore genes was strongly reduced in *spaA-* cells, while expression of many spore stage genes was absent. Chromatin immunoprecipitation (ChIP) of a SpaA-YFP gene fusion showed that (pre)spore gene promoters bind directly to SpaA, identifying SpaA as a transcriptional regulator. SpaA dependent spore gene expression required PKA *in vivo* and was stimulated *in vitro* by the membrane-permeant PKA agonist 8Br-cAMP. The PKA agonist also promoted SpaA binding to (pre)spore promoters, placing SpaA downstream of PKA. Sequencing of SpaA-YFP ChIPed DNA fragments revealed that SpaA binds at least 117 (pre)spore promoters, including those of other transcription factors that activate some spore genes. These factors are not in turn required for *spaA* expression, identifying SpaA as the major trancriptional inducer of sporulation.

## Introduction

Most free-living protists differentiate individually into dormant cysts or spores when challenged by environmental stress. In the Dictyostelia this transition evolved into a multicellular life style that culminates into the formation of fruiting bodies that carry the spores aloft. cAMP crucially regulates this process both as a secreted signal inducing chemotactic aggregation of starving amoebas and expression of aggregation genes and prespore genes, and as an intracellular messenger acting on PKA to induce maturation of spore and stalk cells. Comparative evolutionary studies revealed that these roles of cAMP are derived from a second messenger role of cAMP in stress-induced encystation in the unicellular amoebozoan ancestor^1^. The adenylate cyclase AcrA and the cAMP phosphodiesterase RegA critically regulate intracellular cAMP levels in *D*. *discoideum*, with RegA activity being controlled by multiple sensor histidine kinases^2^. In *D*. *discoideum* the sensor histidine kinases detect signals within the fruiting body that regulate the transition from motile amoebas into encapsulated spore and stalk cells at the correct time and place. AcrA, RegA and PKA also control encystation of solitary amoebas^3,4^ and sensor histidine kinases are abundant in their genomes^5^, acting here as likely sensors for environmental stimuli.

At present, we have limited information of processes that occur downstream of PKA. Three transcription factors, CudA, BzpF and SrfA were shown to regulate prespore and spore-specifc gene expression. CudA promotes expression of prespore genes in the slug^6^, while BzpF and SrfA act later to induce subsets of spore-specific genes. *BzpF* null mutants initially form walled spores, but these spores disintegrate while still in the spore head^7^. *SrfA* null mutants have defects in morphogenesis and form spherical instead of elliptical spores, with diminished viability^8,9^. The expression of both *BzpF* and *SrfA* is upregulated by PKA, but they are not targets for phosphorylation by PKA^7,10^. Loss of a fourth transcription factor, StkA causes prespore cells to transdifferentiate into stalk cells^11^. Transdifferentiation does not occur in mutants that cannot activate PKA in prespore cells, such as *acbA-*, *acrA*-, and psA::PKARm, which just leave the prespore cells amoeboid and unencapsulated^2,12^, indicating that StkA regulates a different developmental choice.

To identify missing components in the sporulation pathway, we used an insertional mutagenesis approach^13^ on cells transformed with a fusion construct of the spore coat gene *cotC* and monomeric red fluorescent protein (mRFP). The screen yielded a mutant that formed fruiting bodies in which prespore cells remained amoeboid. The defective gene, *spaA*, was a nuclear protein that bound directly to and activated the promoters of known spore genes in a PKA dependent manner. Sequencing of immuno-precipitated chromatin fragments cross-linked to SpaA-YFP revealed over 300 putative target genes for SpaA.

## Results

### Identification of a transcription factor essential for spore formation

**Figure 1:**
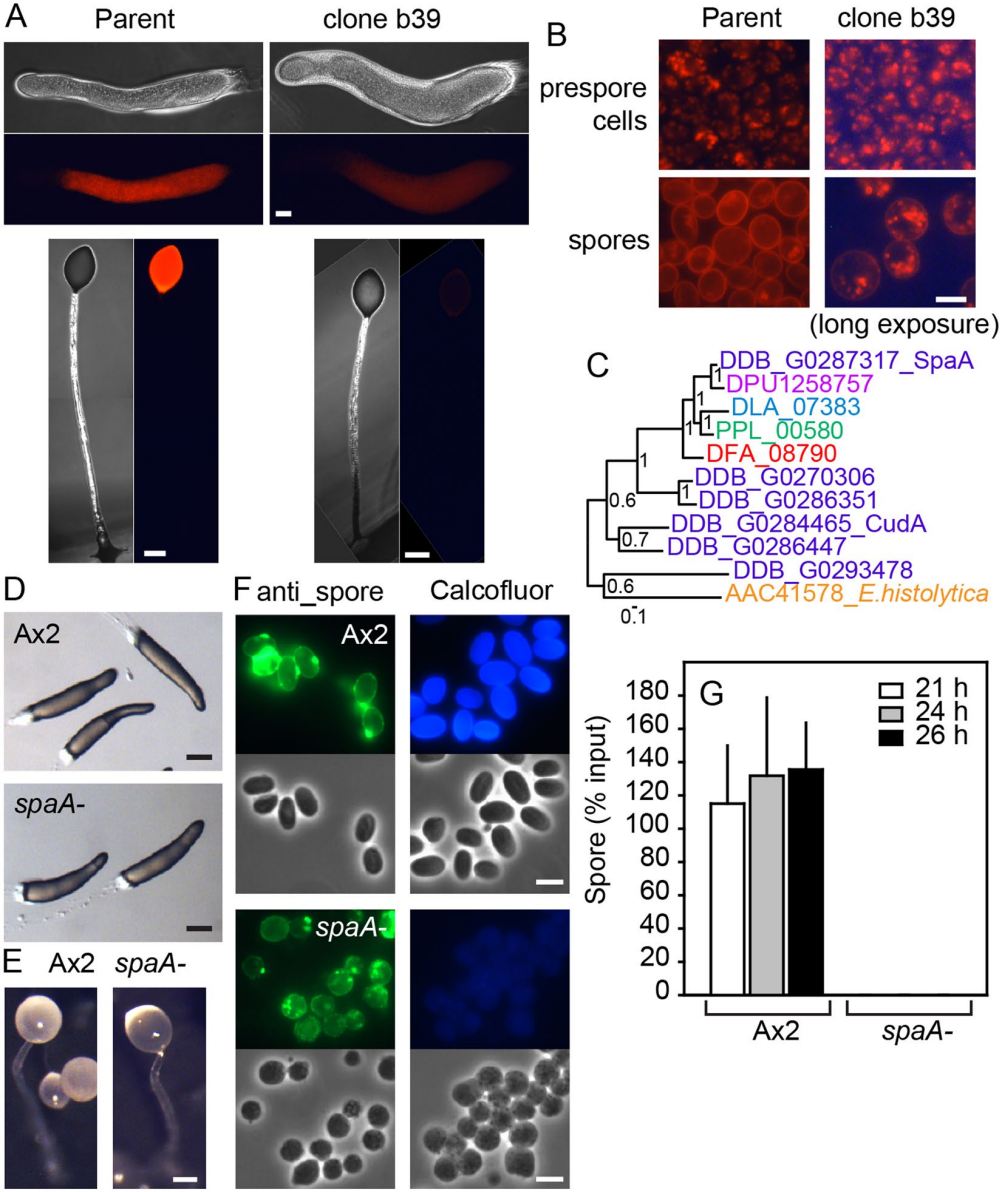
Identification of SpaA by REMI mutagenesis and validation by gene knock-out. (**A**) *REMI mutant phenotype*. Wild-type Ax2 cells, transformed with cotC-mRFP were subjected to REMI mutagenesis and a clone, b39, with reduced red fluorescence in slugs and fruiting bodies was isolated. Bar: 50 µm. (**B**) *Individual cells*. Prespore cells and spores harvested from Ax2 and b39 slugs and fruiting bodies, respectively, were photographed under epifluorescence with longer exposure for the b39 clone. Bar: 3 µm. (**C**) *Phylogeny of mutant gene*. B39 was defective in a CudA-like nuclear factor, SpaA. SpaA sequence was aligned with all *D*.*discoideum* CudA-like proteins (DDB_G prefix), an *Entamoeba histolytica* CudA (AAC41578) and the closest homologs to SpaA in the *Dictyostelids D*. *purpureum* (DPU1258757), *D*. *lacteum* (DLA_07383), *Polysphondylium pallidum* (PPL_00580) and *D*. *fasciculatum* (DFA_08790). A well conserved segment of the alignment (AA184-AA589 of SpaA) was subjected to Bayesian phylogenetic inference^51^. The tree is rooted at midpoint and posterior probabilities of the nodes are indicated. Colour coding of species names: red, green, blue violet: major groups 1, 2, 3 and 4 of Dictyostelia, respectively, amber: non-dictyostelid Amoebozoa. (**D**,**E**) *Recapitulated spaA- mutant*. A *spaA* knock-out was generated by homologous recombination (see Supplementary Fig. S2). Wild type Ax2 and *spaA-* cells were distributed on non-nutrient agar and photographed after slugs (D) and fruiting bodies (E) had formed. Bar: 100 µm. (**F**). *Spore morphology*. Ax2 and *spaA-* spores were fixed and stained with anti-spore antibodies (left) or Calcofluor (right), and photographed under phase contrast (bottom) or epifluorescence (top). Bar: 5 µm. (**G**) *Sporulation efficiency*. Fruiting bodies were developed from 3 × 10^6^ cells plated on 1 cm^2^ filters. Filters were vortexed with 0.1% Triton-X100 after 21 h when fruiting bodies had formed and at two later time points. After spore counting, the percentage of spores relative to the number of plated cells was calculated. Mean and SD of three independent experiments.

To identify unknown components in the *D*. *discoideum* sporulation pathway, we performed REMI mutagenesis and screened for mutants with defects in prespore or spore differentiation. To visualise these processes, we generated a strain in which the red fluorescent protein mRFPmars^14^ was fused to the 3′ end of the spore gene *cotC*. CotC protein accumulates in Golgi-derived prespore vesicles (PSVs) in slugs and is exocytosed at spore maturation to become incorporated in the spore coat^15^ and this was also the case for cotC-mRFP (Fig. 1B). REMI mutagenesis of Ax2/cotC-mRFP yielded a mutant, b39, with strongly reduced RFP fluorescence in the slugs and spore heads (Fig. 1A). Its prespore cells still showed weakly stained PSVs, but PSV exocytosis in spores was incomplete (Fig. 1B).

Sequencing of the amplified region that flanked the plasmid insertion site showed that insertion occurred at a DpnII site in gene DDB_G0287317, which encodes a cudA-like transcription factor.

We named the gene *SpaA* for spores absent A. The *D*. *discoideum* genome contains five genes homologous to *cudA*, and a *cudA* homolog is also present in *Entamoeba*^16^. SpaA has homologs in genomes representative of the four major groups of Dictyostelia^17–19^, which are more closely related to SpaA than to the other *D*.*discoideum cudA*-like genes (Fig. 1C), and are therefore likely SpaA orthologs. Alignment of SpaA with CudA and the SpaA orthologs in other Dictyostelia shows that the conserved core region of the *Entamoeba* and *Dictyostelium* CudA is present in all SpaA orthologs (Supplementary Fig. S1). Two 3- and 5 amino-acid sections of this region, which, when mutated in *Entamoeba* CudA, reduce DNA binding^16^, are well conserved in all SpaA orthologs. We assume from its homology to CudA that SpaA is also a DNA binding protein.

To confirm that the sporulation defective phenotype of the REMI mutant was due to a lesion in *spaA*, we generated a *spaA* null mutant by deleting part of the SpaA DNA binding region (Supplementary Fig. S2). The *spaA-* cells developed normally into migrating slugs and fruiting bodies (Fig. 1D,E). However, as observed with REMI mutant b39, the spore heads of the *spaA-* mutant were more “glassy” than the “milky” wild-type spore heads, and contained only round and phase dark cells (Fig. 1E,F). Antibodies against intact spores stain PSVs in prespore cells, or the wall of spores^20^. However, the *spaA-* spores showed both peripheral staining and granular staining inside the cells, most likely PSVs. Wild-type spores become strongly fluorescent when stained with Calcofluor, a reagent that interacts with cellulose, but the *spaA-* spores were only weakly fluorescent (Fig. 1F). It therefore appears that *spaA-* cells pass partially through PSV exocytosis, but never form cellulose containing spore walls. Unlike wild-type spores, the *spaA-* spores lysed when incubated with detergent, regardless of the time passed after fruiting bodies had matured (Fig. 1G).

### SpaA localization and cell autonomous function

Data retrieved from genome-wide developmental gene expression profiles^21^ show that *spaA* transcription is strongly upregulated from 8–24 h of development and that transcripts are highly prespore-enriched (Supplementary Fig. S3A,B). To analyse localization of SpaA protein in both cells and multicellular structures, *spaA*, inclusive of its promoter, was fused to YFP and transformed into *spaA-* cells. A protein of the expected size of 92 kD was detected by Western analysis (Supplementary Fig. S3C) and the expression of spaA::SpaA_YFP in *spaA-* cells rescued spore formation, indicating that SpaA*_YFP* is functional (Supplementary Fig. S3D). Conform to the prespore enrichment of *spaA* mRNA, SpaA_YFP protein was expressed in the prespore region of slugs (Supplementary Fig. S3E). SpaA_YFP co-localized with the DNA stain DAPI in the nuclei of cells (Supplementary Fig. S3F) at all developmental stages where the protein was expressed. This localization is consistent with a role of SpaA in gene regulation.

SpaA expression in prespore nuclei suggests that SpaA has a cell-autonomous role in spore differentiation. To test this, we mixed the *spaA-* REMI mutant, which carried the cotC:mRFP marker, at different ratios with wild-type cells. The mRFP positive *spaA-* cells were readily incorporated into the spore head, but never differentiated into normal spores (Supplementary Fig. S3G). Using the recapitulated *spaA-* strain, we determined the contribution of detergent resistant spores to chimeric fruiting bodies as percentage of the initial number of plated cells (Supplementary Fig. S3H). Wild-type Ax2, developed alone, produced more than 100% spores, which is probably due to prespore cell division during development^22^. Percentages of viable spores in chimeric structures were proportional to the percentage of wild type cells in the starting mixture. We developed these spores as clones and confirmed by examination of spores in their fruiting bodies that they all derived from Ax2. Evidently, the *spaA-* mutant has a cell-autonomous defect in spore formation.

### Expression of post-aggregative genes in spaA-

**Figure 2:**
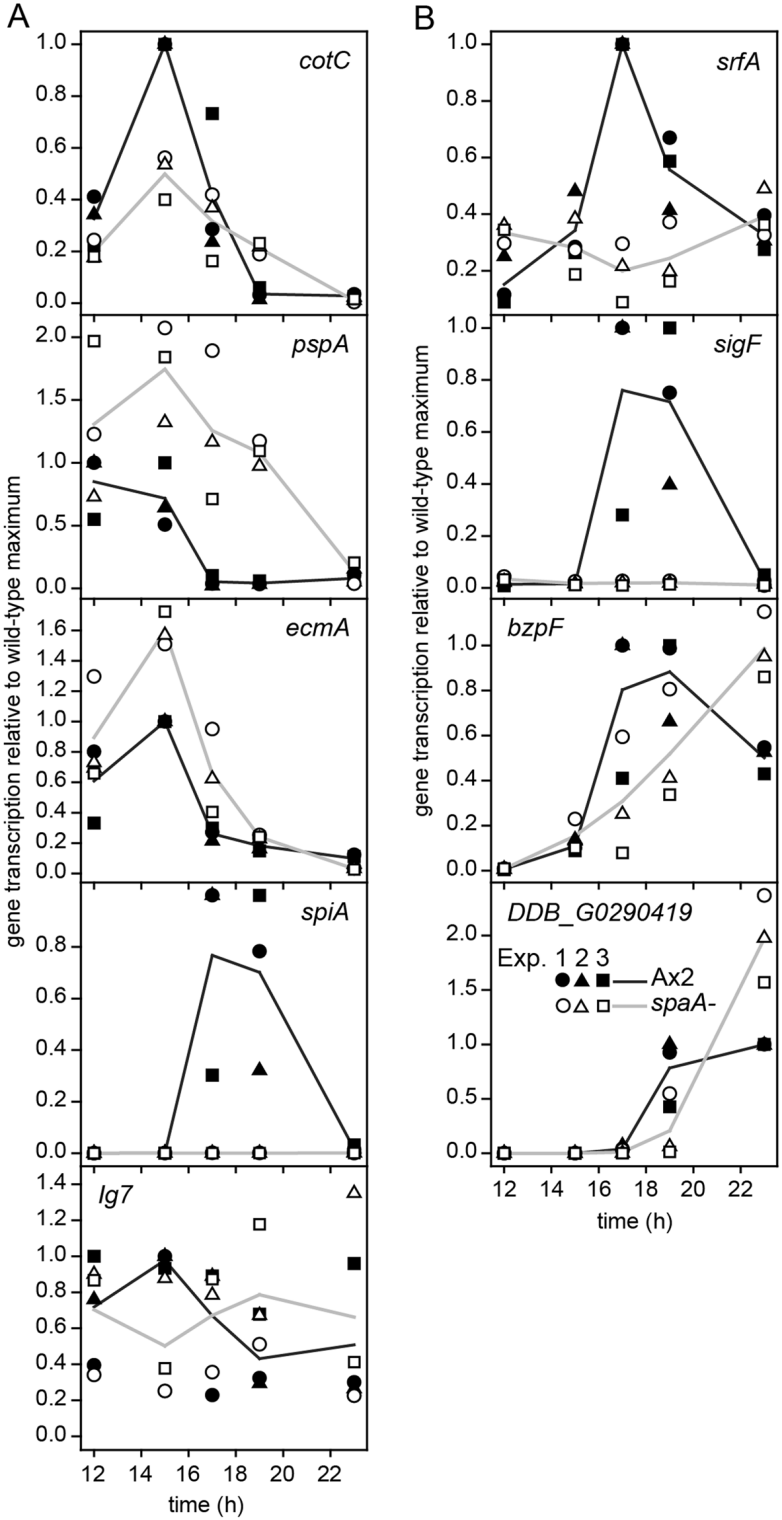
Expression of cell type specific genes in *spaA-* cells. Wild-type Ax2 and *spaA-* cells were plated for development to fruiting bodies (24 h), and total RNA was isolated at 2 h intervals, starting at mounds (12 h). mRNA levels for the prespore genes *cotC* and *pspA*, the prestalk gene *ecmA*, the spore gene *SpiA*, the constitutively expressed gene *Ig7* (**A**) the transcription factors *srfA* and *bzpF* and their respective targets *sigF* and *DDB_G0290419* (**B**) were measured by qRT-PCR. Data are expressed as fraction of the highest expression obtained with Ax2 for each experiment. Because the timing of peak expression differs between experiments, data from three experiments are plotted separately. Black and grey lines connect the mean values of the three experiments for Ax2 and *spaA-*, respectively.

The homology of SpaA with the transcription factor CudA suggests that SpaA is also a transcription factor. We therefore compared developmental expression of the prespore genes *pspA*^23^ and *cotC*^15^, the spore gene *spiA*^24^ and the prestalk gene *ecmA*^25^ between wild-type and *spaA-* cells, using quantitative reverse transcription-PCR (qRT-PCR). *EcmA*, *cotC* and *pspA* are highly expressed in wild-type slugs at 15 h of development, with expression decreasing during fruiting body formation. Expression of *ecmA* and *pspA* is 30–50% increased in *spaA-* cells, while *cotC* expression is 50% decreased (Fig. 2A). *spiA* is optimally expressed during mid-culmination in wild-type, but not at all in *spaA-*, indicating that SpaA is particularly important for spore gene expression.

Two transcription factors, SrfA and BzpF are required for full spore viability^7,8^. Transcriptomic analyses identified *sigF* and *spiA* as target genes for SrfA, and *DDB_G0290419* as a target gene for BzpF^7,8,26^. *BzpF* is upregulated in spores, while *srfA* is expressed in both prestalk and prespore cells, with expression being upregulated in maturing spores. Figure 2B shows that *bzpF* and its target *DDB_G0290419* are still expressed in the *spaA-* mutant, although expression seems delayed. *SrfA* is similarly expressed in *spaA-* and wild-type cells at 12–14 h. However, the transient increase at 18 h of wild-type *srfA* expression is absent in *spaA-*. Expression of the SrfA target *sigF* is lost in *spaA-*. SpaA appears to be essential for expression of SrfA and SrfA-dependent genes during sporulation, whereas it is not critically required for expression of BzpF and one of its target genes.

### PKA activation does not rescue the sporulation defect of *spaA-*

**Figure 3:**
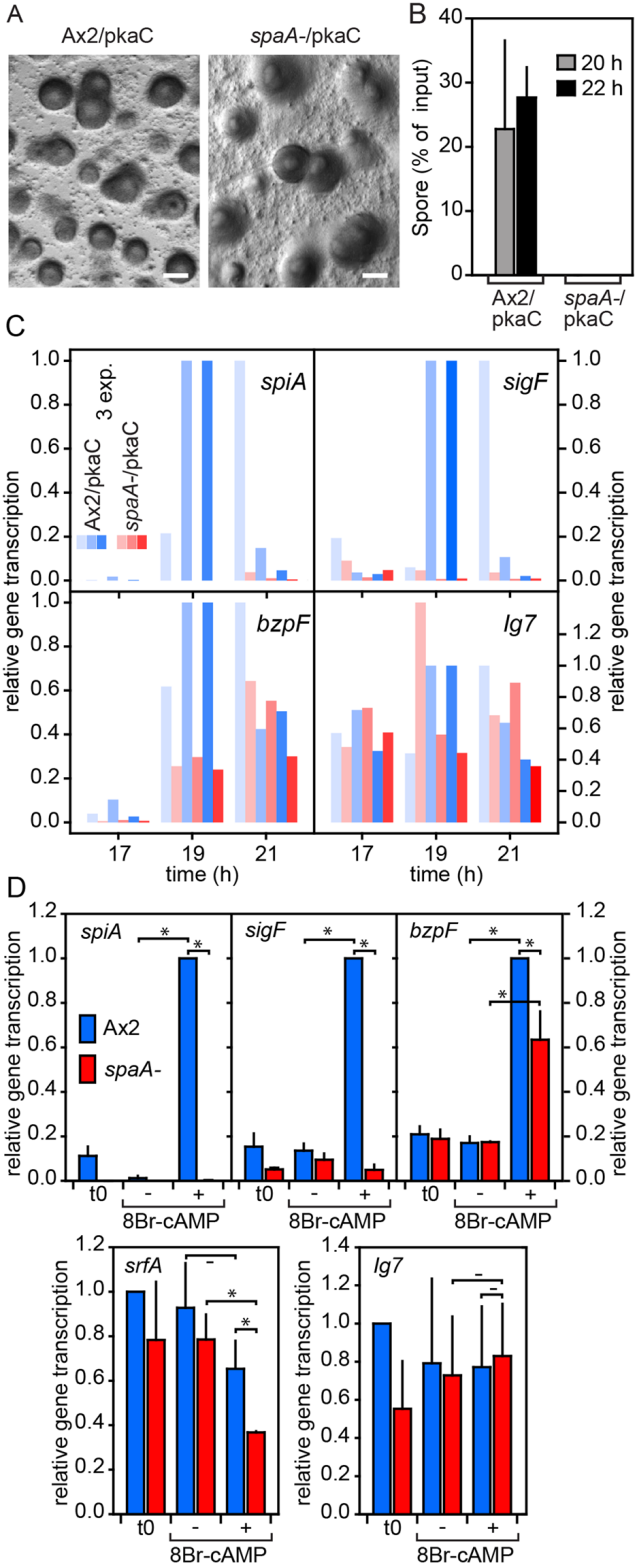
PkaC overexpression and 8Br-cAMP treatment of *spaA-*. (**A**) *Mound arrest*. Ax2 and *spaA-* cells, transformed with an A15::pkaC-YFP construct, were developed for 24 h (when wild-type has formed fruiting bodies, see Fig. 1E) and photographed. Bar: 300 µm. (**B**) *Spore formation*. The efficiency of sporulation of A15::pkaC-YFP transformed AX2 and *spaA-* cells was measured as described for Fig. 1G. (**C**) *PkaC effects on gene expression*. Cells were developed for 16 h at 12 °C and 1, 3 and 5 h at 22 °C. At 3 and 5 h spores have formed in Ax2/A15::pkaC-YFP. RNA was isolated and transcription of *spiA*, *sigF*, *bzpF* and *Ig7* was determined by qRT-PCR. Values are expressed as fraction of the highest expression obtained with Ax2/A15::pkaC-YFP. Results of three individual experiments are shown. (**D**) *8Br-cAMP effects on gene expression*. Ax2 and *spaA-* early culminants were dissociated, resuspended to 10^7^ cells/ml and incubated with or without 15 mM 8Br-cAMP. RNAs were isolated at t0 and after 2 h of incubation, and levels of *spiA*, *sigF*, *bzpF*, *srfA*, and control *Ig7* transcripts were determined by qRT-PCR. Data are expressed as fraction of the highest expression obtained with Ax2, and means and SD of 3 experiments are shown. Significant differences between some treatments as determined by a rank sum test are indicated (*P < 0.01; -: P ≥ 0.05).

The transition of prespore cells into spores requires PKA^12,27^ and to assess whether SpaA acts either upstream or downstream of PKA, or in a parallel pathway, we tested whether overexpression of the PKA catalytic subunit (PkaC) could restore spore formation in *spaA-*. A construct in which *pkaC* was fused N-terminally to the actin15 promoter and C-terminally to YFP was transformed into wild-type and *spaA-* cells, and clones with high expression, as evident from Western blots, were selected. Ax2 cells with high expression of A15::pkaC_YFP showed precocious sporulation and mound arrest (Fig. 3A,B), while lower expressing clones arrested as slugs as reported earlier^28^. *SpaA-*/A15::pkaC_YFP cells also showed mound arrest, but no detergent resistant spores were formed (Fig. 3A,B). Transcription of the SpaA-dependent genes *spiA* and *sigF* was also not restored (Fig. 3C) and *bzpF* expression was still delayed in *spaA-*/A15::pkaC_YFP cells, as was the case in *spaA-* (Fig. 2).

To validate that active PKA cannot restore the sporulation defect of *spaA-*, we acutely activated PKA in early culminant wild-type and *spaA-* cells with the membrane-permeant PKA activator, 8Br-cAMP. Figure 3D shows that 8Br-cAMP effectively induces *spiA and sigF* expression in wild-type, but not in *spaA-*. *BzpF* induction by 8Br-cAMP is about 40% reduced in *spaA-*. Complex expression of *srfA* during development is regulated by alternative promoters that generate mRNAs with different exons in the 5′UTR. The most distal promoter activates *srfA* expression during spore maturation, and its activity is enhanced when 8Br-cAMP is added to the substratum^9,10^. We found that 8Br-cAMP decreased *srfA* expression from its full endogenous promoter in both Ax2 and *spaA-*. The control gene *Ig7* was similarly expressed in Ax2 and *spaA-*, irrespective of treatment with 8Br-cAMP.

### SpaA binds to the spore-specific promoters in a PKA dependent manner

**Figure 4:**
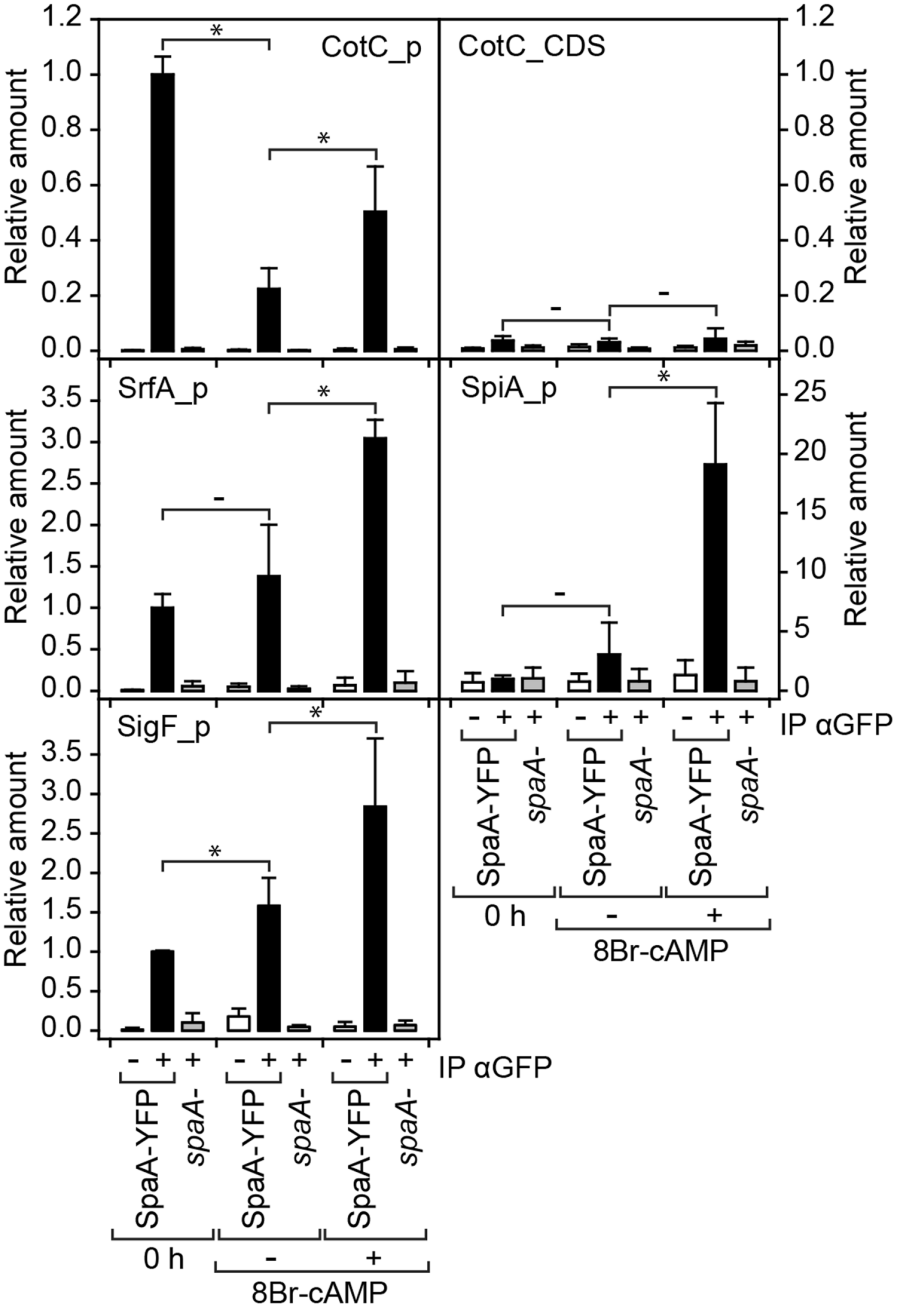
Chromatin immuno-precipitation with SpaA-YFP. *SpaA-* and *spaA-*/SpaA-YFP early culminants were dissociated and incubated for 1 h with or without 10 mM 8Br-cAMP. After crosslinking to protein, chromatin was sheared and immuno-precipitated with or without αGFP antibody. The presence of *cotC*, *srfA*, *spiA* and *sigF* promoter sequences in the immuno-precipitates was determined by qPCR using promoter specific primers (Supplementary Table S2), or *cotC* coding sequence (CDS) specific primers as control. Amounts of amplified products are normalized to amounts obtained from diluted total cell lysate and expressed as fraction of DNA amplified from promoter regions at 0 h in antibody treated SpaA-YFP expressing cells. Means and SD of 3 experiments. Significant changes (P < 0.05) between some treatments are indicated by asterisks.

To investigate whether SpaA binds to spore gene promoters, we performed chromatin immuno-precipitation, using the SpaA_YFP fusion protein expressed from the *spaA* promoter in *spaA-* cells (Supplementary Fig. S3C). The construct was expressed under hygromycin selection, which requires only a single copy number of the vector, to minimize artefacts due to overexpression. *SpaA-*/SpaA_YFP and *spaA-* control cells were developed to early culminants and incubated for 1 h without and with 8Br-cAMP to enhance expression of spore genes. After protein-DNA crosslinking and DNA shearing, cell lysates were immuno-precipitated with or without αGFP antibody, and the presence of target gene promoters in the immuno-precipitate was determined by qPCR. For *srfA*, PCR primers were designed against promoter sequences essential for spore-specific *srfA* expression. Figure 4 shows that over 10-fold more *cotC*, *srfA*, *spiA* and *sigF* promoter sequences were amplified from *spaA-*/SpaA_YFP preparations incubated with αGFP antibodies than without antibody, and that untransformed *spaA-* cells showed almost no pull-down of promoter sequences. For *srfA*, *spiA* and *sigF*, promoter pull-down was lower at t = 0 h and without 8Br-cAMP treatment, but *cotC* promoter pull-down was highest at the start of the experiment, in agreement with the earlier developmental expression of *cotC* (Fig. 2A). qPCR with primers close to the  3′ end of the *cotC* coding region showed only some amplification in the antibody treated *spaA-*/SpaA_YFP samples, which could result from some larger DNA fragments surviving shearing. Overall, the experiment shows that SpaA binds directly to the promoter regions of its target genes, consolidating its role as transcriptional regulator.

### ChIPseq analysis identifies more than hundred SpaA target genes

We performed ChIPseq to identify all genes that are regulated by SpaA. *spaA-/SpaA_YFP* cells were developed into culminants. DNA libraries with fragments of ~300 bp were prepared from ChIPed DNA in three separate experiments and subjected to 75 bp paired-end sequencing. After mapping reads to the genome, the read pairs that mapped concordantly were used to identify peaks with read counts that significantly exceeded read counts from control libraries. Such peaks were annotated to the gene with the closest start codon. The three experiments yielded a total of 2036 peaks, of which 815 were shared between experiments 2 and 3 and 345 between all three experiments (Supplementary Table S1). This low overlap is largely due to low ChIP efficiency in the first experiment. The 815 and 345 peaks mapped to 640 and 312 genes, of which respectively 364 and 216 were protein coding genes. Most of the other genes were transposons or retrotransposons. In contrast to the protein coding genes, where peaks mapped upstream of the start codon, the (retro)transposon genes mostly had peaks downstream of the start codon (Supplementary Fig. S4) and the greater majority was not developmentally transcribed (Supplementary Fig. S5). They were therefore not analysed further. Of the 389 and 227 peaks annotated to protein coding genes 84 and 88% were in the 5′ intergenic region with a median distance of −434 bp and −415 bp from the start codon, respectively (Supplementary Fig. S4E,H).

**Figure 5:**
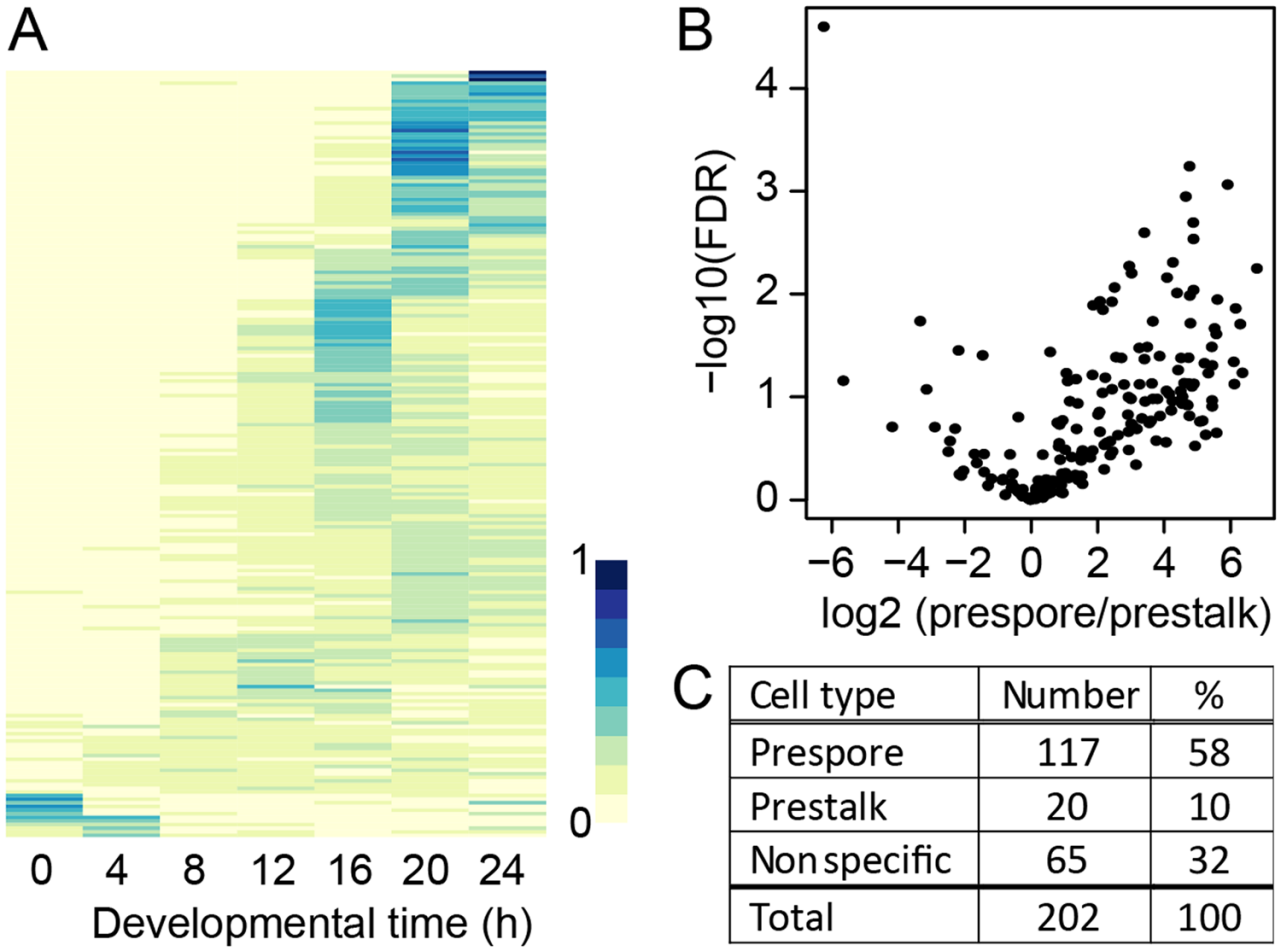
Developmental expression pattern and cell-type specificity of SpaA targets. (**A**) *Developmental expression*. Heatmap of developmental transcript levels of the common set of protein coding genes that bind SpaA in three ChIPseq experiments. (**B**,**C**) *Cell-type specificity*. Volcano plot (**B**) of transcript enrichment between prespore and prestalk cells of the genes binding to SpaA. (**C**) Genes that were over 2-fold enriched in either cell-type or that fell below this threshold (non-specific) were counted and data were recalculated as percentages. Normalized read counts for the developmental time points and prestalk and prespore cell fractions were retrieved from^21^. (See also Supplementary Table S1 and Figures S5 and S6).

Most of the 216 protein-coding genes, ChIPed by SpaA in all three experiments, showed highest expression in either the slug or fruiting body stage (Fig. 5A), and 58% and 10% were over 2-fold enriched in prespore or prestalk cells, respectively. Not all genes detected in the published time course also showed expression in the cell-type specificity experiment^21^. The percentage of prespore- or prestalk- enriched genes is therefore calculated from the 202 genes with read counts in the cell-type specificity experiment (Fig. 5C).

The larger set of 364 protein-coding genes, common to experiments 2 and 3, showed similar developmental regulation (Supplementary Fig. S6), but was 9% less enriched in prespore cells (Supplementary Fig. S5). However, since this set had 49 more prespore-specific genes than the 216 set, likely to be valid SpaA targets, it was included in further analysis. The number of prespore-enriched genes common to all three experiments, or experiments 2 and 3 only, are 117 and 166, respectively (Supplementary Fig. S5J), which, considering the (pre)spore-specificity of SpaA itself, sets the limits to the number of likely SpaA targets. Cluster analysis based on developmental expression indicated that genes upregulated after 12 h of development showed higher statistical significance of read peaks (lower q-values) and more pronounced prespore enrichment than clusters that were expressed earlier or constitutively, or that were downregulated in development (see supplementary spreadsheet SupData1, sheets 3 and 4). Since SpaA is itself only expressed after aggregation, this suggests that the early expressed and down-regulated genes bind SpaA fortuitously.

Gene ontology (GO) analysis revealed that both the 216 and 364 sets of SpaA targets show in the category Biological Process strongest overrepresentation of GO terms associated with “sporulation”, “cell wall assembly” and “fruiting body development”, while in the category Cellular Component GO terms associated with the plasma membrane and spore wall are most overrepresented (SupData1, sheets 5 and 6). Among the SpaA target genes with known or predicted functions are many spore coat genes (*cotA-C*, *pspB*,*D*, *psvA*, *DDB_G029139*2), an expansin, *expl3*, the poly-glycosyltransferase *pgtB*^29^, the UDP-glucose 4-epimerase *galE*^30^, the GlcNAc transferase *gnt11* and the cellulose synthase, *dcsA*^31^, all involved in spore wall synthesis, and the water channels *aqpA*^32^ and *wacA*^33^ (Table 1 and SupData1, sheet 7). Ten SpaA target genes are transcription factors of which three *spaA*, *cudA*^16^ and *srfA*^34^ are involved in sporulation, while loss of *stkA* causes prespore cells to transdifferentiate into stalk cells^11^. Some protein kinases and phosphatases are SpaA targets. Most are prespore-enriched, but only *lkb1* is known to facilitate sporulation^35^. In addition, SpaA binds to 20 other genes with signalling roles, of which *abcG6*, *abcF2*, *abcH2*^36^, *gadA*^37^, *nox*C^38^ and *tagB*^39^ are required for spore formation or full spore viability (Table 1).

**Table 1:** Spore physiology- and signalling genes that bind to SpaA.

spore wall/ membrane	transcription factor	protein kinase/ phosphatase	various signalling genes	
*aqpA*	bzpL	abkA	5NT	gpaB
cotA	*cudA*	DDB_G0267686	abcD2	*noxC*
cotB	DDB_G0287317	DDB_G0286841	*abcF2*	patB
cotC	DDB_G0293478	glkA	*abcG6*	phyA
*dcsA*	dimB	*lkb1*	*abcH2*	pldB
DDB_G0291392	gtaL	pakG	arfA	secG
expl3	gtaP	pXi	arpB	*tagB*
galE	mybE	smg1	culA	yelA
gnt11	mybN		darA	
pgtB	*spaA*	**protein phosphatase**	DDB_G0269128	
pspB	*srfA*		DDB_G0279727	
pspD	*stkA*	DDB_G0271350	DDB_G0282093	
pspE		ptpC	DDB_G0284619	
pspG			dmtA	
psvA			*gadA*	
wacA			glpD	

Data are summarized from a listing complete with peak enrichment and q-values of the 364 SpaA target genes common to ChIPseq experiments 2 and 3 (see supplementary spreadsheet SupData1, sheet 7) Underlined: enriched in prespore cells; italics: null mutant has sporulation defect.

### Validation of SpaA targets and cross-regulation of other transcription factors

**Figure 6:**
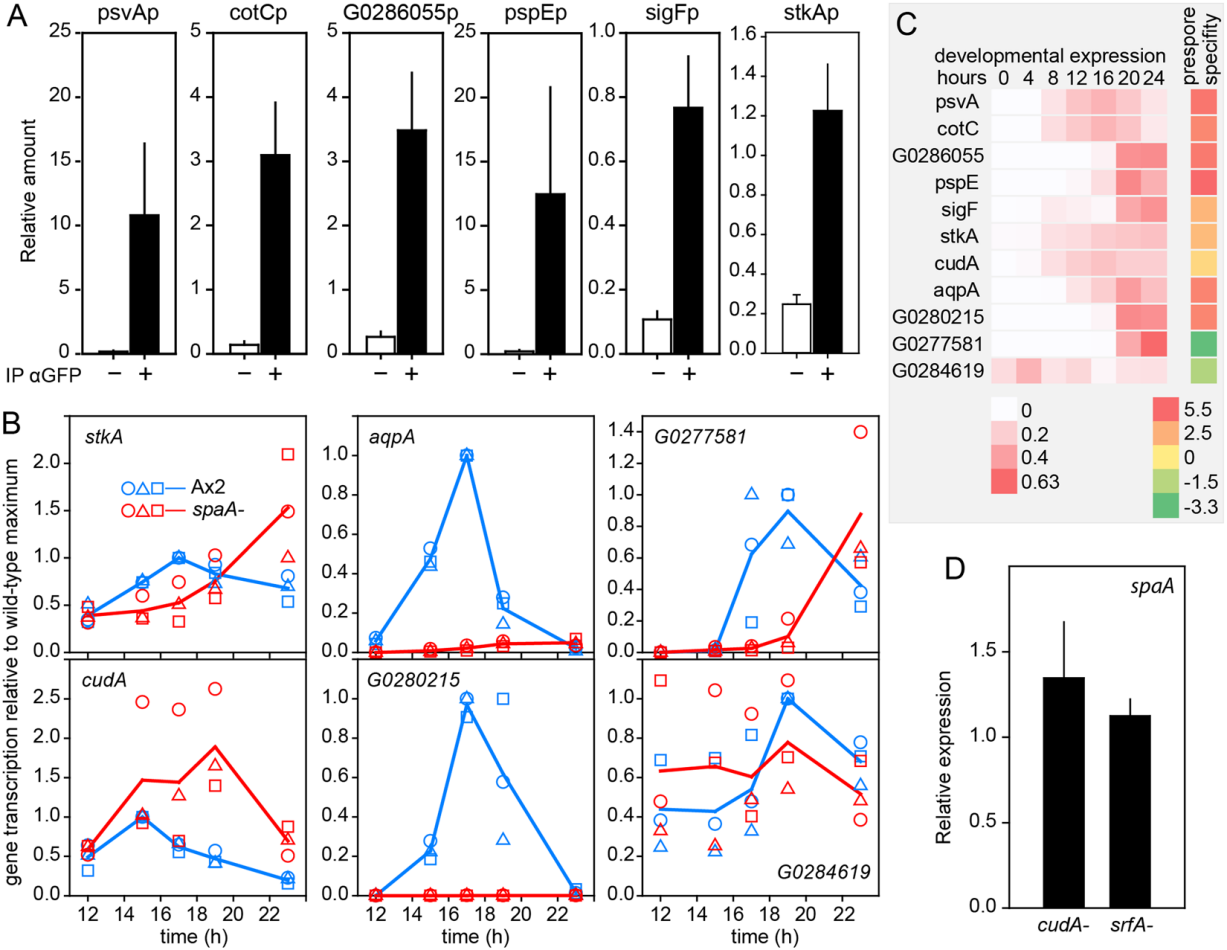
Validation of SpaA targets identified by ChIPseq. (**A**) *SpaA target immunoprecipitation*. Total chromatin and immuno-precipitates prepared with or without αGFP antibody from *spaA-*/spaA-YFP culminants for the ChIP-seq experiments (see Methods) were subjected to qPCR with promoter specific primers (Supplementary Table S2) to determine the presence of promoter regions of the *psvA*, *cotC*, *DDB_G0286055*, *pspE* and *sigF* genes. Amounts of amplified products are expressed relative to amounts obtained from diluted total chromatin. Means and SD of 3 independent experiments. (**B**) *Developmental expression*. mRNA levels of putative SpaA targets were measured by qRT-PCR during the final 12 h of Ax2 and *spaA-* development as in Fig. 2. Data are expressed relative to the highest expression obtained in Ax2, and data from three experiments are plotted separately with blue symbols for Ax2 and red symbols for *spaA-*. Blue and red lines connect the mean values of the three experiments for Ax2 and *spaA-*, respectively. (**C**) *Expression profiles and prespore specificity*. Heat maps of standardized expression profiles (read counts expressed as fraction of the read count sum of all developmental time points) and prespore/prestalk cell-type enrichment (^2^log fold-change) of the genes investigated in panels A and B. Data are retrieved from two high-throughput RNA sequencing experiments of *D*.*discoideum* AX4 developmental time courses and purified prestalk and prespore cells^21^. (**D**) *SpaA expression in cudA- and srfA-*. Null mutants in *cudA*^6^ and *srfA*^8^ and their respective parents Ax2 and Ax4, were developed for 16 hr in the dark untill standing slugs had formed. RNA was isolated and expression of *spaA* was determined by qRT-PCR. Expression in mutant cells was normalised to expression in the parental strain. Mean and SD of 2 experiments.

To validate the ChIPseq approach, the promoter pulldown of 6 putative SpaA targets in SpaA-ChIPed chromatin was tested by qPCR. Figure 6A shows that all 6 promoters were amplified. As expected for SpaA targets, *CotC* and *sigF* are underexpressed in *spaA-* (Fig. 2) and we investigated this also for *stkA* and 5 other putative targets. The prespore genes, *aqpA* and *DDB_G0280215*, expressed after 16 h, are also down-regulated, but *cudA* and *stkA* which are less prespore-enriched and transcribed earlier, are still expressed in *spaA-* (Fig. 6B). Expression of prestalk genes DDB_G0284619 and *DDB_G0277581* are not significantly altered or delayed in *spaA-*, respectively. Apparently, late expressed prespore genes are more stringently regulated by SpaA than early expressed or non-prespore genes.

We further assessed whether SpaA shares targets with other transcription factors that promote sporulation. For SrfA 41 putative targets were detected by interrogation of microarrays^26^. BzpF has 24 putative and 15 confirmed targets^7^ and for StkA 8 targets were identified by differential display^40^. CudA has only 2 confirmed targets, *cotC*^16^ and the prestalk gene *expl7*. SpaA shares no targets with BzpF, and only 5 targets with SrfA, amongst which *sigF* and *spiA* (Table 2). There were 2 and 1 SpaA targets in common with StkA and CudA, respectively, but these numbers may be underestimates, due to the low number of identified targets for either StkA or CudA. Because SrfA is down-regulated in *spaA-* cells (Fig. 2B), SpaA affects SrfA targets mostly by inducing expression of SrfA itself. This is not the case for CudA and StkA, which are still expressed in *spaA-* (Fig. 6B). We also investigated *spaA* expression in *cudA-* and *srfA-* mutants (the *stkA-* mutant is not available anymore). However, it appeared that neither CudA nor SrfA are required for *SpaA* expression (Fig. 6D).

**Table 2:** Target overlap between SpaA and other transcription factors.

	Known downstream genes	SpaA targets	
		Common to exp. 1–3^*1^	Common to exp. 2 & 3^*2^
SpaA		216	364
SrfA	41	2	5
StkA	8	1	2
BzpF	24	0	0
CudA	2	1	1

The identity of the shared target genes is listed in Supdata1, sheet 8.

(*1) number of genes annotated to peaks observed in all 3 experiments.

(*2) number of genes annotated to peaks common to experiments 2 and 3.

## Discussion

A sporulation deficient mutant was isolated from a REMI screen for mutants defective in prespore gene expression. The mutant partially exocytosed its prespore vesicles, but was incapable of synthesizing the spore wall and its mutated gene, *spaA*, encoded a deeply conserved cudA-type transcription factor. Similar to *acbA-*, *acrA*-, and psA::PKARm mutants that cannot activate PKA in prespore cells^2,12^, *spaA-* mutants leave prespore cells amoeboid in the spore head, suggesting that SpaA acts downstream of PKA. This was validated by observations that SpaA binds to spore promoters in a PKA-dependent manner and that *spaA-* mutants lack PKA-induced spore gene expression. Using purified TAP-tagged SpaA and YFP-tagged PKA-C, we were however unable to detect direct phosphorylation of SpaA by PKA (unpublished results), indicating that there must be at least one phosphorylated intermediate.

High throughput sequencing of DNA immunoprecipitated by SpaA-YFP detected 216 to 364 protein coding genes with significant binding to SpaA in their 5′ intergenic region. The gene set contained many spore coat genes and was enriched in gene ontology terms associated with sporulation, spore wall assembly and fruiting body development. About half of the genes were upregulated in the slug and fruiting body stage and showed highest expression in prespore cells. Genes with earlier expression and/or lacking prespore specificity were not or less stringently down-regulated in the *spaA-* mutant (Fig. 6B,C), suggesting that the affinity of their promoters for SpaA was fortuitous or less relevant for their overall regulation.

**Figure 7:**
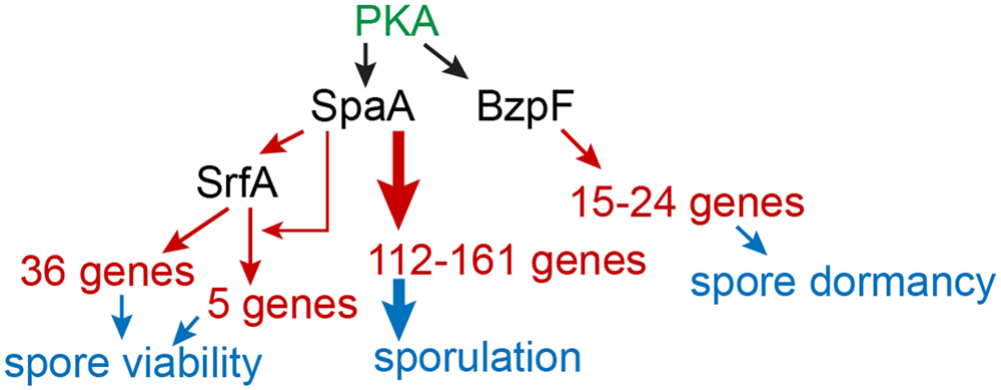
Hierarchy of transcription factors that regulate *Dictyostelium* sporulation. SrfA upregulates 41 genes required for spore viability and elliptical spore shape, and is itself upregulated by SpaA, which controls >100 spore genes, amongst which 5 are co-regulated with SrfA. BzpF upregulates 15–24 genes that act to prevent precocious germination. All three transcription factors act downstream of PKA.

Among the SpaA target genes was the transcription factor *srfA*, which also regulates spore gene expression. Five out of the 41 SrfA target genes were also SpaA targets, while SpaA is not itself an SrfA target. This indicates that besides the more than 100 spore genes that are directly activated by SpaA, SpaA controls even more genes by inducing SrfA expression. This places SpaA at the top of a hierarchy that controls spore differentiation (Fig. 7). CudA and StkA, two other transcription factors that are involved in spore differentation^6,11^ as well as MybE and DimB with roles in prestalk differentiation^41^ are also putative targets for SpaA (Table 1). However, there are up till now only a few target genes known for either of these factors, making it difficult to assess to what extent they contribute to the genes that are ultimately regulated by SpaA. BzpF, also required for sporulation, and its target genes do not require SpaA for expression. However, whereas *spaA-* makes no walled spores, *bzpF-* makes spores with cellulose-rich walls, which disintegrate after a few days^7^. This suggests that BzpF acts later by activating genes required for dormancy.

Many SpaA target genes encode proteins with different roles in assembly of the spore coat^42^, which were shown to be coordinately regulated by PKA activation^43^. Overall, the pronounced effects of *spaA* deletion on sporulation and the vast number of genes under SpaA control hallmark this transcription factor as the key regulator of *Dictyostelium* sporulation.

## Methods

### Cell culture

*Dictyostelium discoideum* Ax2 was cultured either in HL5 axenic medium (Formedium, UK) or on SM agar plates in association with *Klebsiella aerogenes*. For development, cells were distributed at 3 × 10^6^ cells/cm^2^ on non-nutrient agar, or on nitrocellulose filters supported by filter pads soaked with DB (1 mM CaCl_2_ and 2 mM MgCl_2_ in 5 mM Na/K phosphate buffer, pH 6.5).

### REMI mutagenesis of cotC-mRFP transformed cells

The *cotC* gene, ranging from nt −743 relative to the start codon to the end of the coding sequence was amplified from Ax2 genomic DNA using primers cotC-f and cotC-r (Supplementary Table S2) and blunt-end cloned into vector pJet1.2 (Thermo Fisher Scientific, Whaltham, MA). The *cotC* fragment was excised with XbaI and BamHI and cloned into the XbaI/BamHI digested plasmid pExp4/ecmO:mRFPmars, replacing the *ecmO* promoter^14^. The resulting *cotC-mRFP* gene fusion was cloned into pExpHyg, a pExp-4(+) vector^44^, in which the neomycin resistance cassette was replaced by the hygromycin resistance cassette from pHygTm(+)/pG7 using XbaI and XhoI. The construct was transformed into Ax2 cells and transformants were selected at 30 µg/ml hygromycin.

For REMI mutagenesis a clonal isolate of AX2/*cotC-mRFP* was transformed with pUCBsrΔBam, linearized with BamHI, and 1 unit of DpnII^13^. Transformants were selected at 10 µg/ml blasticidin and clonally plated with *K*. *aerogenes*. Developing clones were inspected under a stereo microscope and a compound fluorescence microscope for defects in slug and fruiting body morphology and/or defects in red fluorescent staining of the prespore and spore regions. The site of plasmid insertion in selected mutants was determined by inverse PCR^45^ using primers Remi-f and Remi-r (Supplementary Table S2). The amplified fragment was sequenced with primer BsrA15r (Supplementary Table S2).

### Recapitulation of the spaA lesion by gene knock-out

To generate an *spaA* knock-out vector, a genomic fragment of the *spaA* coding region was amplified with primers spaA-f1 and spaA-r1 (Supplementary Table S2) and cloned into vector pJet1.2. The *spaA* region, located 5′ to the internal BamHI site at nucleotide 1155, was replaced with a fragment amplified with spaA-f1 and spaA963r (Supplementary Table S2), deleting a region between 964 and 1155, while maintaining the BamHI site. The blasticidin resistance cassette of pLPBLP^46^ was excised with SmaI and blunt-end ligated into the BamHI digested and filled-in spaA-KO vector. The vector was linearised with EcoRI and transformed into Ax2 cells. Transformants were selected at 10 µg/ml blasticidin and diagnosed for *spaA* gene disruption by two PCR reactions (Supplementary Fig. S2).

A *D*. *discoideum* genomic fragment, containing the *spaA* coding region and 2.6 kb upstream of the start codon, was amplified with primers spaA-f2 and spaA-r2 (Supplementary Table S2), digested with SalI and EcoRI and cloned into vector pExp4. YFP was next inserted at the 3′ end of *spaA* using EcoRI and XhoI. The *spaA-YFP* fragment was excised using SalI and XhoI and ligated into SalI/XhoI digested pExpHyg. The construct was transformed into Ax2 and *spaA-* cells, and transformants were selected at 30 µg/ml hygromycin. For Western analysis, slug stage cells were lysed in SDS-sample buffer, proteins were separated on 4–12% polyacrylamide gels (Thermo Fisher Scientific, Whaltham, MA), transferred to nitrocellulose and probed with anti-GFP antibody (Roche Applied Science, Penzberg, Germany), followed by HRP-conjugated anti-mouse antibody. YFP-positive bands were detected using SuperSignal West Pico Chemiluminescent Substrate (Thermo Fisher Scientific, Whaltham, MA).

### Immunostaining

Spores were fixed in 85% methanol and incubated with rabbit-anti-spore antibodies^47^, diluted 1:10.000 in PBS with 5% bovine serum albumin (BSA) and with 1:2000 diluted Alexa488 conjugated goat-anti-rabbit-IgG (Thermo Fisher Scientific, Whaltham, MA). Cellulose was stained with 20 µg/ml Calcofluor White (Sigma-Aldrich, St. Louis, MO). For whole mount staining, structures developed on polytetrafluoroethylene membrane (Merck Millipore, Billerica, MA), were fixed with 50% and 100% methanol, successively, and stained with 1:2000 diluted mouse-anti-GFP antibody and 1:2000 diluted Alexa-Fluor594 conjugated anti-mouse antibody (Thermo Fisher Scientific, Whaltham, MA). Structures were mounted in the presence of 3 µM DAPI and imaged using a Leica LP2 confocal microscope.

### RNA analysis by qRT-PCR

RNA was isolated from about 10^7^ cells using the RNAeasy mini kit (Qiagen, Hilden, Germany) and transcribed into cDNA with the ImProm-II Reverse Transcription System (Promega, Fitchburg, WI) or the sensiFAST cDNA synthesis kit (Bioline, London, UK) in the experiments of Fig. 2 or Fig. 6, respectively. Quantitative PCR (qPCR) was performed using PerfeCTa SYBR Green SuperMix (Quanta biosciences, Beverly, MA) with technical duplicates and the primers listed in Supplementary Table S2.

### Chromatin immunoprecipitation (ChIP)

Cells, developed into early culminants, were incubated with and without 10 mM 8Br-cAMP for 1 hr. Cells were fixed with 1% formaldehyde in PBS at a density of 5 × 10^7^ cells/ml for 10 min, and for another 15 min after addition of glycine to a final concentration of 125 mM. Cells were washed with PBS, followed by RET buffer (50 mM TRIS, pH 8.0, 150 mM NaCl, 0.1% (w/v) SDS, 1% (v/v) NP-40, 0.5% (w/v) sodium deoxycholate, 2 mM EDTA, 0.05% (v/v) Triton X-100 in 50 mM Tris, pH 8), and resuspended in RET buffer containing cOmplete EDTA-free protease inhibitor cocktail (Roche Applied Science, Penzberg, Germany). Chromatin was sheared by sonication (Branson Sonifier 150) with 3 pulses of 30 s at setting 6 to produce fragments of 100 to 1000 bp. The lysate was centrifuged at 16000 × g for 10 min, and 30 µl of the supernatant was kept as total DNA. For immunoprecipitation, 350 µl supernatant was incubated overnight at 4 °C with anti-GFP antibody and ProteinG-Dynabeads (Thermo Fisher Scientific, Whaltham, MA). Beads were washed sequentially with RET buffer, TTST buffer (150 mM NaCl, 1% Triton X100, 0.1% SDS, 2 mM EDTA, 0.05% Tween20 in 50 mM Tris-Cl, pH 8) and LiCl buffer (250 mM LiCl, 1% sodium deoxycholate, 1% NP40, 1 mM EDTA in 10 mM TRIS, pH 8), and eluted with 1% SDS and 1 mM EDTA in 50 mM Tris-Cl, pH 7.5. The eluate and the total DNA was supplemented with 1/10 volume of 3 M NaCl and incubated at 65 °C overnight to reverse the cross-link, treated with 100 µg/ml proteinase K and 100 µg/ml RNase, and purified on a QIAquick column (Qiagen, Hilden, Germany). The amount of immuno-precipitated DNA was determined by qPCR using the primers listed in Supplementary Table S2 and PerfeCTa SYBR Green SuperMix with technical duplicates. Total DNA was 50x diluted before qPCR to obtain a similar amplification rates as ChIP DNA. In cases where DNA fragments were undetectable and failed to get valid Ct values, the amount was assumed to be zero.

### ChIP-seq

ChIP was performed as described above, using a *spaA-/*spaA-YFP mixture of slugs to late culminants, except that samples were sonicated 5 × 30 s at setting 8 to obtain smaller fragments. 20 µl lysate was kept as total DNA, and immunoprecipitation was performed with anti-GFP antibody on 1.1 ml lysate. Control precipitation without antibody was performed from 350 µl lysate. Aliquots of total chromatin and immunoprecipitates were used to analyse the presence of promoter sequences by qPCR (Fig. 6), with the amount of amplified product in immuno-precipitates adjusted by sample volume and expressed as relative amount to 30x diluted total DNA. Sequencing libraries were constructed from ChIPed DNA and 30x diluted total DNA as follows: DNA ends were repaired with Klenow polymerase and phosphorylated with T4 polynucletide kinase. After addition of an A-overhang, pre-annealed ChIP-seq Top and Bottom adaptors (Supplementary Table S3) were ligated onto the DNAs. Between steps, DNA was purified with Agencourt AMPure XP beads (Beckman Coulter, Brea, CA). DNA was amplified with the forward and reverse ChIPseq primers (Supplementary Table S3) and fragments <800 bp were isolated by gel electrophoresis. Each sample library was verified by Qubit 3.0 and Agilent 2200 TapeStation before pooling. Libraries were pooled to allow 6 samples per run on the NextSeq500 platform. Paired-end Illumina sequencing was performed using the Mid-Output v2, 150 cycle kit.

Sequence reads were mapped against the *D*. *discoideum* genome (v.13–05–2009) using Bowtie2^48^. Reads mapped concordantly within 700 bp were used to call peaks by MACS2^49^ with a q-value cut off of <0.01, allowing a 200 bp shift in positions of peak summits between experiments. Peaks were annotated to the gene with the closest start codon, using ChIPpeakAnno^50^. Gene ontology (GO) enrichment was determined using the Amigo tool at the GO consortium (www.geneontology.org/) with a threshold cutoff of p ≥ 0.05.

### Data availibility

ChIPseq data have been deposited in the ArrayExpress database at EMBL-EBI (www.ebi.ac.uk/arrayexpress) under accession number E-MTAB-6000. All knockout mutants and plasmid constructs have been deposited at the *Dictyostelium* Stock Center http://dictybase.org/StockCenter/StockCenter.html.

## Electronic supplementary material

Supplementary information

Dataset 1
