# Supplementary material for: The transcription factor Spores Absent A is a PKA dependent inducer of Dictyostelium sporulation
Source: Sci Rep. 2018 Apr 27;8:6643. doi: 10.1038/s41598-018-24915-w (PMC5923282; doi:10.1038/s41598-018-24915-w)
Supplement: Supplementary file 1 — Supplementary information [file 41598_2018_24915_MOESM1_ESM.pdf]

SUPPLEMENTARY INFORMATION

The transcription factor Spores Absent A is a PKA dependent inducer of *Dictyostelium* sporulation

Yoko Yamada[1], Andrew Cassidy[2] and Pauline Schaap[1]*

Supplementary figures S1-S6, supplementary tables S1-S3

Contents

Figure S1. Alignment of SpaA orthologs and homologs. 2
Figure S2. Knockout of *spaA* gene. 3
Figure S3. Developmental regulation, localization and cell-autonomous requirement of *spaA*. 4
Figure S4. Location of read peaks from three ChIPseq experiments, relative to start codons. 5
Figure S5. Cell type specificity of SpaA target genes identified in three ChIPseq experiments. 6
Figure S6. Developmental regulation of SpaA targets common to experiments 2 and 3 7
Table S1. Significantly enriched read count peaks and corresponding genes from three ChIPseq experiments 7
Table S2. Oligo nucleotide primers for cloning, RT-qPCR and ChIP analysis 8
Table S3. Oligonucleotide primers used for ChIPseq 9
Supplementary References 9

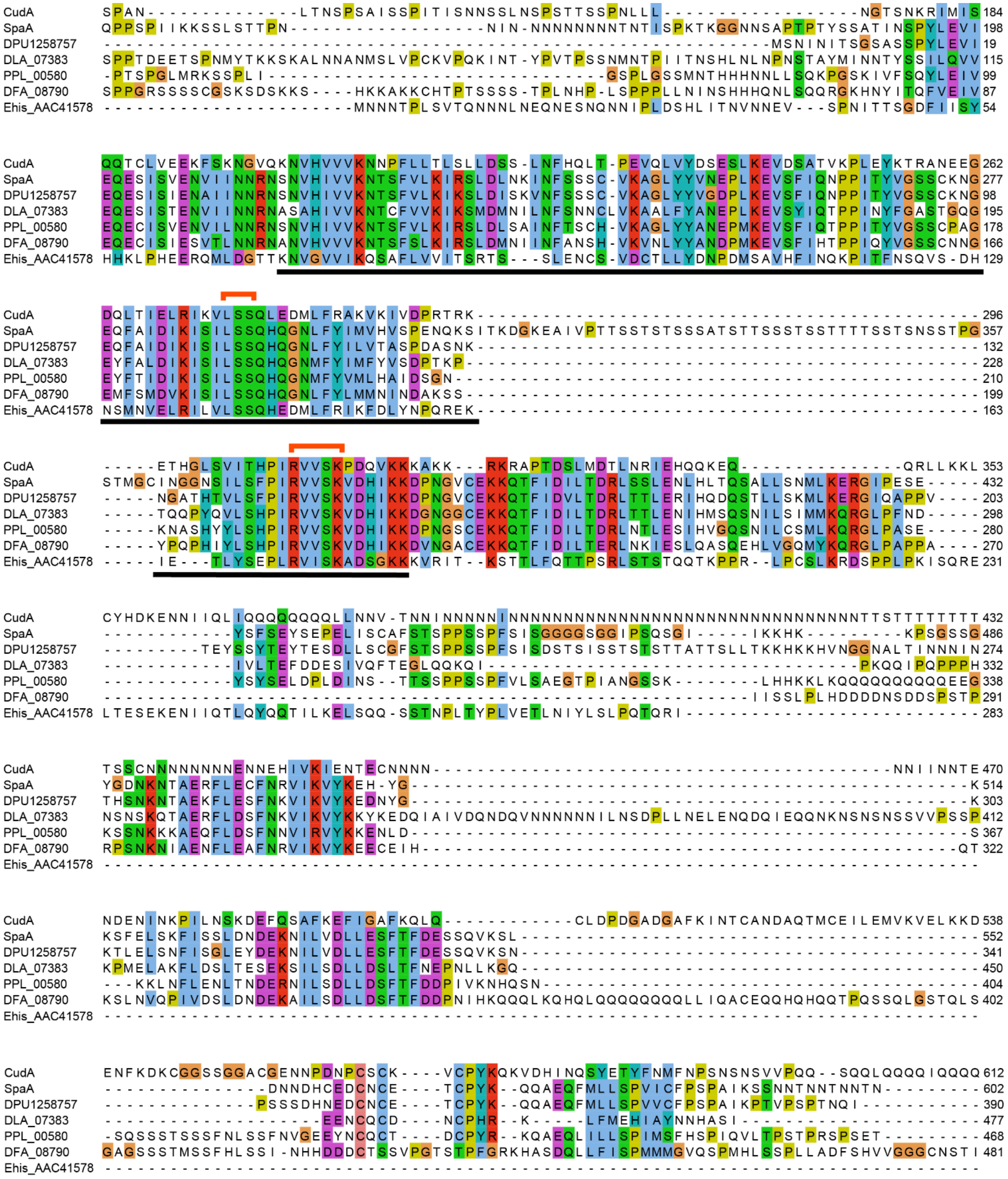

Figure S1. Alignment of SpaA orthologs and homologs.

D.discoideum SpaA and its orthologs from *D. purpureum, D. lacteum, Polysphondylium pallidum* and *D. fasciculatum* were aligned with *D. discoideum* CudA and an *E. histolytica* CudA homolog, which was used previously to identify two regions (red brackets) that contain essential residues for DNA binding [1]. A 120 amino acid core region of homology that is shared between the *D. discoideum* and *E. histolytica* CudAs is underlined in black. The poorly conserved N- and C-termini are not shown.

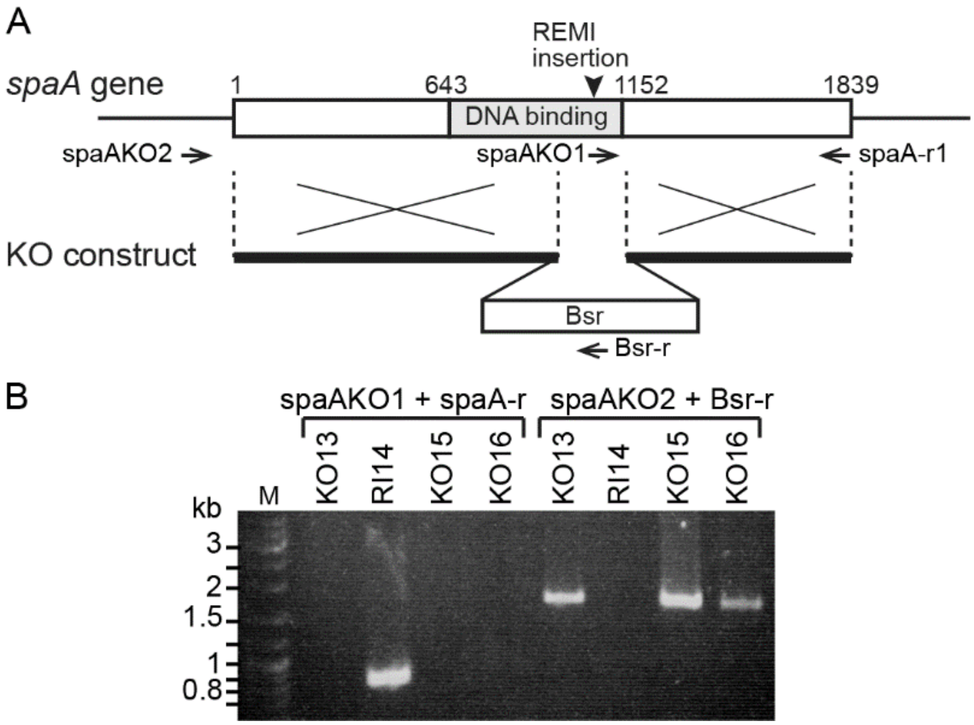

Figure S2. Knockout of *spaA* gene.

A. Diagram of the *spaA* genomic region and the knockout construct with the blasticidin resistance cassette (Bsr) replacing part of the conserved DNA binding region. Primers spaAKO1 and spaA-r1 amplify a 0.8 kb fragment from random integrants (RI), whereas primers spaAKO2 and Bsr-r amplify a 1.8 kb fragment from successful homologous recombination with *spaA* (KO).

B. To identify *spaA* knockout clones, genomic DNA was prepared from clonal isolates of wild-type cells transformed with the KO construct, and analysed by PCR using the primers mentioned above. M: DNA marker.

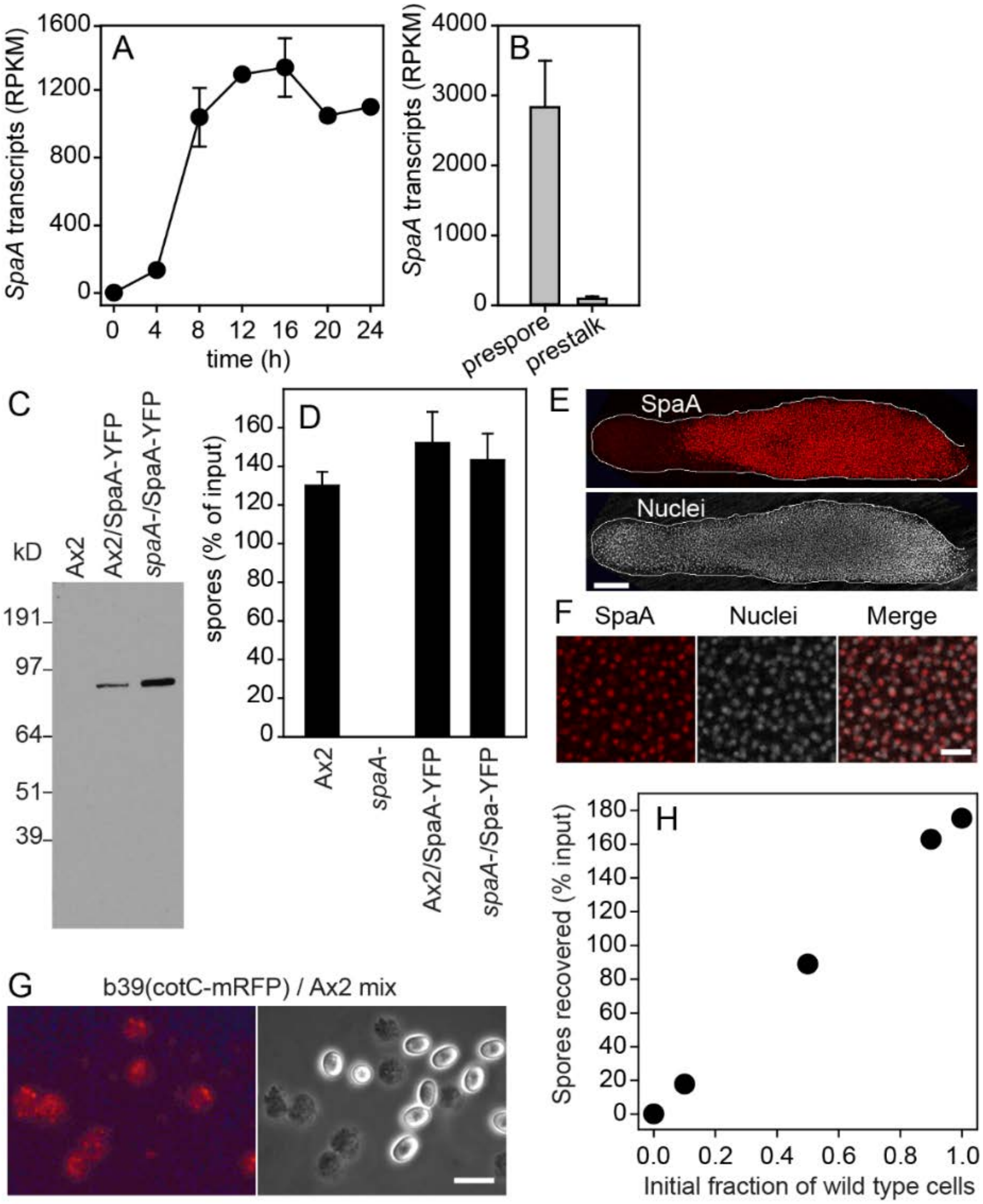

Figure S3. Developmental regulation, localization and cell-autonomous requirement of *spaA.*

A. *Developmental regulation and pattern of spaA gene expression.* Normalized read counts for *spaA* (DDB_G0287317) transcripts were retrieved from two published high throughput RNA sequencing experiments [2]. Averaged RPKM (reads per kilobase per million mapped reads) of the two experiments are presented with standard deviations for a developmental time course (A) and prestalk and prespore cells isolated from *D.discoideum* AX4 slugs (B).

C. Expression of a SpaA-YFP fusion protein. Western blot of Ax2 and *spaA*- cells, transformed with a construct of the *spaA* gene inclusive of its promoter and fused at the 3'end to YFP. Together with untransformed Ax2, transformants were developed into slugs, dissolved in sample buffer and gel fractionated. After transfer, bands were visualized with αGFP antibody and chemiluminescence.

D. *Restoration of sporulation*. Fixed numbers of Ax2, *spaA*- and SpaA-YFP transformed cells were plated on filters as described in the legend to Fig. 1G and the number of detergent-resistant spores in fruiting bodies was determined and expressed as percentage of input. Means and SD of 3 experiments.

E/F. *SpaA localization*. Slugs of Ax2/*spaA-YFP* cells were fixed and stained with αGFP antibody and Alexa594 conjugated anti-mouse antibody, and counterstained with DAPI. Structures were photographed at low (E, bar: 100 µm) and high (F, bar: 10 µm) magnification, using a confocal microscope.

G/H. *Chimeric development.* G. Cells of REMI clone b39, harbouring cotC-mRFP, were mixed at a 1:4 ratio with Ax2 cells and developed into fruiting bodies. Spores were collected and photographed under phase contrast or epifluorescence. Bar: 20 µm. H. *SpaA*- and wild-type cells were mixed at different ratios, and $3x10^6$ cells were developed to fruiting bodies on filters. The percentage of TritonX-100 resistant spores, relative to input cell number, was determined, and plotted against the fraction of wild type cells in the original mixture. The average of 2 independent experiments is shown.

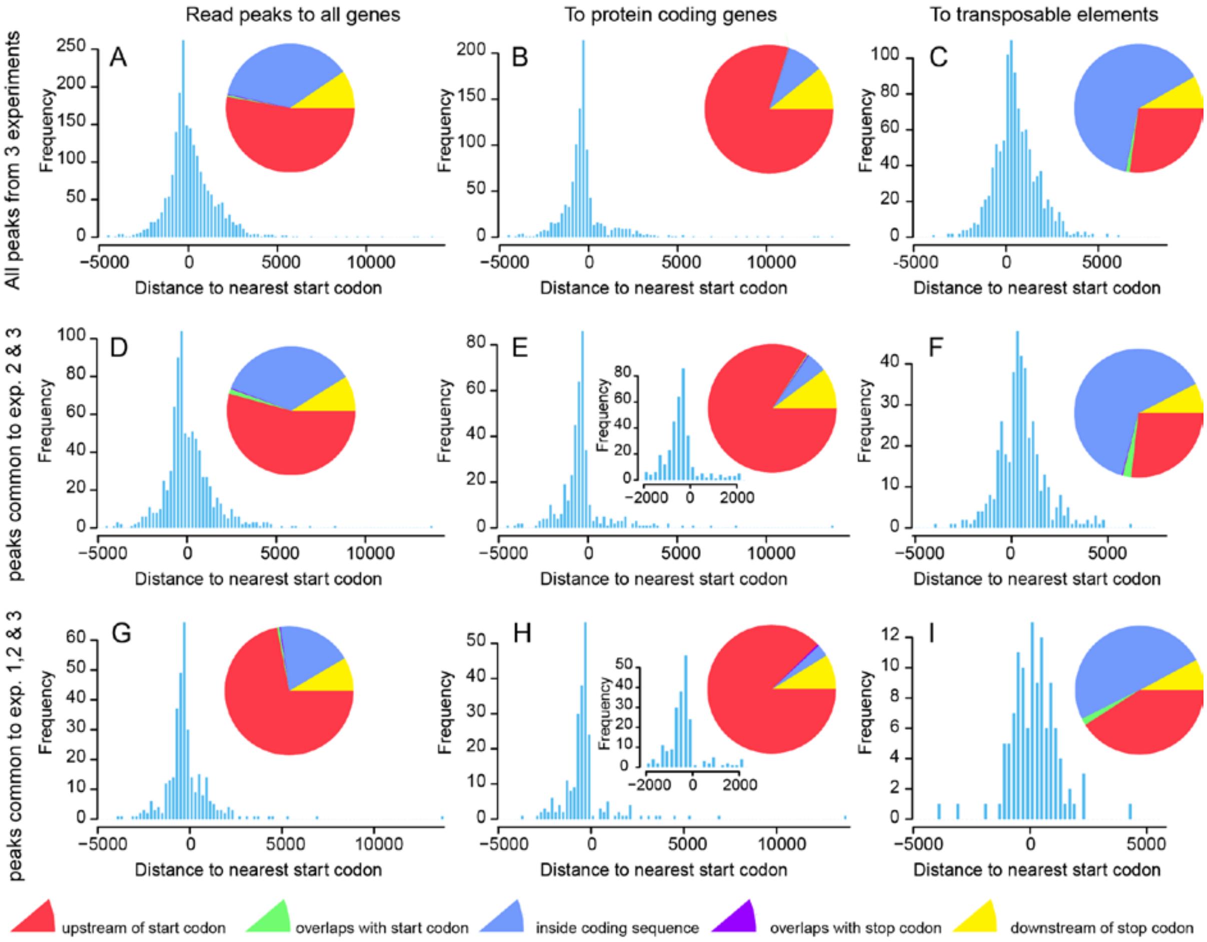

Figure S4. Location of read peaks from three ChIPseq experiments, relative to start codons.

A-I. Histograms of peak distances relative to the start codon of the gene to which the peaks were annotated, and pie charts showing the proportions of peaks with locations up- or downstream or inside coding sequences, or overlapping with start or stop codons. A-C. Peaks observed in all three experiments. D-F. Peaks common to experiments 2 and 3. G-I. Peaks common to all three experiments. A,D,G. Peaks annotated to all genes, B,E,G. Peaks annotated to protein coding genes. C,F,I. Peaks annotated to (retro)transposons. E,H. The region between -2 kb and 2 kb is enlarged in the inset. 5 peaks at a distance >25 kb are not shown. The data show that over a third of all peaks were annotated to retrotransposons, but that such peaks were mostly located inside the coding sequence. The peaks annotated to protein coding genes were mostly located upstream of the start codon. The peaks annotated to protein coding genes had on average much better statistical support than those annotated to retrotransposons (SupData1.xlxs, sheet 1).

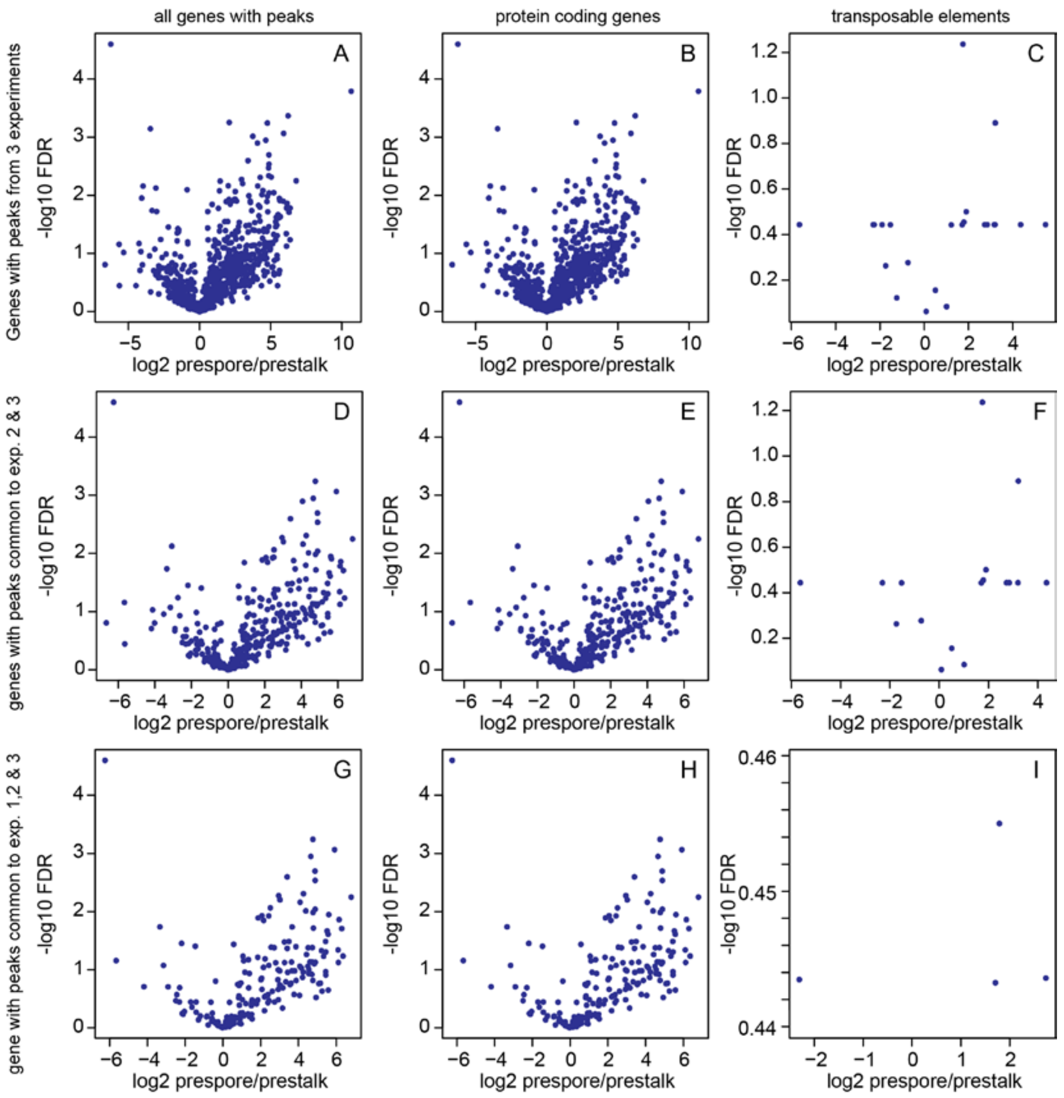

J

over 2-fold cell-type enrichment	All 3 experiments						Common to experiments 2 & 3						Common to experiments. 1, 2 and 3					
	all genes		prot. cod.		transp. el.		all genes		prot. cod.		transp. el.		all genes		prot. cod.		transp. el.	
	n	%	n	%	n	%	n	%	n	%	n	%	n	%	n	%	n	%
prepore	368	46	355	45	13	57	176	50	166	49	10	59	120	58	117	58	3	75
prestalk	118	15	111	14	7	30	49	14	45	13	4	24	21	10	20	10	1	25
non-enriched	319	40	316	40	3	13	129	36	126	37	3	18	65	32	65	32	0	0
total	805	100	782	100	23	100	354	100	337	100	17	100	206	100	202	100	4	100

Figure S5. Cell type specificity of SpaA target genes identified in three ChIPseq experiments. Transcript enrichment across prestalk and prespore cells of the genes annotated with read peaks from all three ChIPseq experiments was retrieved from existing RNAseq experiments [2] and presented as Volcano plots. A-C. Genes with peaks in all three experiments. D-F. Genes with peaks common to experiments 2 and 3. G-I. Genes with peaks common to all three experiments. A,D,G. All genes with peaks, B,E,G. Protein coding genes with peaks. C,F,I. (Retro)transposon genes with peaks. Note that very few of the (retro)transposon genes, which make up about 1/3rd of all annotated genes, express transcripts in the slug stage. J. Numbers and percentages of genes with prespore- and prestalk enriched or non-enriched transcripts for the three gene sets and the three experimental data combinations presented in plots A-I. Note that the total number of genes in the three sets is lower than those listed in Table S1. This is due to the fact that transcripts for some of the *SpaA* target genes were not detected in the RNAseq experiment [2]. The cell-type specificity data for all individual genes are summarized in SupData1, sheet 2.

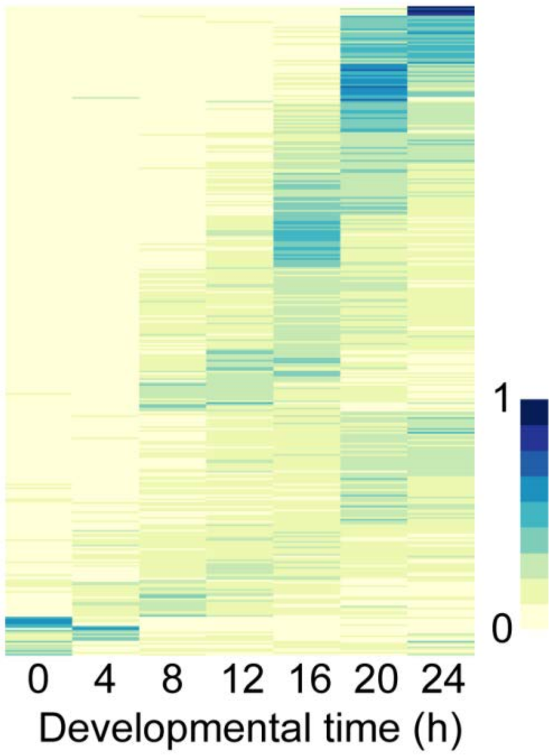

Figure S6. Developmental regulation of SpaA targets common to experiments 2 and 3
Heatmap of developmental transcript levels of the set of protein coding SpaA targets common to ChIPseq experiments 2 and 3. Normalized read counts for the developmental time points were retrieved from [2] and are expressed as fraction of the sum of all time points for each gene.

Table S1. Significantly enriched read count peaks and corresponding genes from three ChIPseq experiments

	all peaks	common to experiments 2&3	common to experiments 1-3
experiment 1	477		345
experiment 2	1217	815	
experiment 3	1599		
annotated to genes		811	342
annotated to unique genes[1]		640	312
to unique protein coding genes		364	216
to unique (retro) transposons		275	96

[1] some genes have more than one read count peak

Table S2. Oligo nucleotide primers for cloning, RT-qPCR and ChIP analysis

**Plasmid construction and REMI mutagenesis**		
cotC-f	TCTAGACCCATACTACATTAAAATATTTGTATATC	
cotC-r	GGATCCATAGTCCCATTCATCATTTGC	
Remi-f	GAATTGCCGCTCCCACATGA	
Remi-r	GCGATTGGTAGTGCAGTTTC	
BsrA15r	GATTTGATGGGATTAATTAATTTGTAATC	
spaA -f1	GAATTCATGATGAGTTATGAACAACAAC	
spaA -r1	CTCGAGTTAAATTGTTACAACAGTATTATTATTTG	
spaA 963r	GGATCCGACAATTGCCTCTTTACCATC	
spaA KO1	CACCAGGATCAACAATGGG	
spaA KO2	CATTCCTCATTCATTCATTCGC	
Bsr-r	GCCGCTCCCACATGATG	
spaA -f2	GTCGACCCTAGTGTAATATCGTACTGTG	
spaA -r2	GAATTCAATTGTTACAACAGTATTATTATTTG	
**RT-qPCR**	forward	reverse
cotC	GAAAGACGTGGTGGTATC	TTGCATCTTGGAAGTCATC
pspA	CGAATATACTACAAACCAATGTACTGTTCA AG	GTGTGGCAGTGATTTTACAAACTCCAC
Ig7	TTACATTTATTAGACCCGAAACCAAGCG	AACAGCTATCACCAAGCTTGATTAGCC
ecmA	CCGTAAACTGTGAATGTGATGACC	GTCTTGGAATCGCAACTATCAGC
spiA	CCAGGAACACCTCAAATGTCATCTGAA	CTAAGTTTTAAATCTTGTTCCCATCTC
bzpF	CACCATCAGGTGTTGCTGATC	ATTCTTTGAAGCGGCTTCAGC
sigF	AGCAGGAAGTTATAACCGTGG	ATGGCTCAGAATAGTTAACTTCC
G0290419	AACAGAAGCTGTTGGTGCTG	CACAAGCATGTTCACATGATTC
srfA	GTAACCCAAATGTTTATCCAATTGG	GGTTTGGAGAAGCTGGTGAGG
spaA	CACCAGGATCAACAATGGG	AACGGTCGGTAAGGATATCG
stkA	GGAACCATCCATAATAGCAAC	CATCACGATAACCACCAACC
cudA	CACGTGCCAATGAAGAAGG	TATGACAGAGAGGCCATGAG
aqpA	GAATTGACTTGGGTGTTCCAC	ACGATTAAGACGACGATCCC
G0280215	CTTGAGAAGAGGTGGTCGTG	AGTCTAGCAGCTCCACCAG
G0277581	CGCAACCTTCCCATGGTTAC	CCACGCATGAAAACGTCACC
G0284619	TGACACCAAGGGAAGGAGG	CAGGAGCAGCATCCATTGAG
**ChIP analysis**	forward	reverse
cotCp	CACCCACACACTAATTTACCC	CATATGCTTGTGTGTTGGGAG
pspAp	TTCATACTGCTGAGCACTCC	ACAGTGGGGTAACATAAGTTG
sigFp	GTGGTAAAGAAAAATGGTGTTGG	CCAGATCACATTAATGCTTTGAG
srfAp	CAACCCCATTAATACATATAAGC	GTTGTTGTACGTGTGGTTAGTG
spiAp	GGTGTGCTTATGTAAATGTGGG	TACTAGATGAGGAAATTAAAGTGTG
cotC CDS	GAAAGACGTGGTGGTATC	CCATCATAGTCACCGTCACG
G0286055p	CACACCACACTTTCAATGTTTC	CTATTTCCGAAAGAAGTGGGTG
pspEp	AATAAAGTTAACCCACATAACCAG	CAAGTTGGGTTGAAGTGGTG
psvAp	GCACACTTTCACCCACTTTG	GGTGAGTGGGTTGGTGTATG
stkAp	CAGTTCAATCTTTGCTAGCAAC	GATCTTATTGGGTTAAGTGTGAG

Table S3. Oligonucleotide primers used for ChIPseq

**Adaptors**	
Top	ACACTCTTTCCCTACACGACGCTCTTCCGATC*T
Bottom	P-GATCGGAAGAGCGGTTCAGCAGGAATGCCGAG
**for PCR**	
forward:	5'_AATGATACGGCGACCACCGAGATCTACACTCTTTCCCTACACGACGCTCTTCCGATC*T_3'
reverse:	
exp1_total	CAAGCAGAAGACGGCATACGAGATAACGTGATGAGATCGGTCTCGGCATTCCTGCTGAACCGCTCTTCCGATC*T
exp1_IP	CAAGCAGAAGACGGCATACGAGATAAACATCGGAGATCGGTCTCGGCATTCCTGCTGAACCGCTCTTCCGATC*T
exp2_total	CAAGCAGAAGACGGCATACGAGATATGCCTAAGAGATCGGTCTCGGCATTCCTGCTGAACCGCTCTTCCGATC*T
exp2_IP	CAAGCAGAAGACGGCATACGAGATAGTGGTCAGAGATCGGTCTCGGCATTCCTGCTGAACCGCTCTTCCGATC*T
exp3_total	CAAGCAGAAGACGGCATACGAGATACCACTGTGAGATCGGTCTCGGCATTCCTGCTGAACCGCTCTTCCGATC*T
exp3_IP	CAAGCAGAAGACGGCATACGAGATACATTGGCGAGATCGGTCTCGGCATTCCTGCTGAACCGCTCTTCCGATC*T

Asterisks (*) indicate phosphorothioate modification

Supplementary References

1. Yamada Y, Wang HY, Fukuzawa M, Barton GJ, Williams JG. A new family of transcription factors. *Development* **135**, 3093-3101 (2008).
2. Parikh A, *et al.* Conserved developmental transcriptomes in evolutionarily divergent species. *Genome Biol* **11**, R35 (2010).
